# Association between Serum Adiponectin and Atrial Fibrillation: A Case-Control Study Stratified by Age and Gender

**DOI:** 10.1155/2021/6633948

**Published:** 2021-02-10

**Authors:** Tongjian Zhu, Zhuo Wang, Songyun Wang, Wei Hu, Hui Chen, Jing Xie, Meng Wang, Kezhong Ma, Hong Jiang

**Affiliations:** Department of Cardiology, Renmin Hospital of Wuhan University, Cardiovascular Research Institute, Wuhan University, Hubei Key Laboratory of Cardiology, Wuhan, Hubei, China

## Abstract

**Background:**

Circulating adiponectin has been suggested to be associated with atrial fibrillation (AF). However, whether the association differs by age and gender remains unknown. We performed a case-control study to evaluate the above association.

**Methods:**

AF patients who underwent 24-hour long-range 12-channel electrocardiogram examination at our center were included in this study, and people with normal sinus rhythm (NSR) were included as controls. All participants underwent echocardiography and heart rate variability tests. Biochemical parameters and adiponectin levels were also evaluated. Receiver operating characteristic (ROC) analyses were used to determine the predictive efficacy of adiponectin for AF, and multivariate logistic regression analysis was performed to evaluate the potential independent predictors of AF.

**Results:**

Overall, 84 patients with AF and 84 people with NSR were included. Serum adiponectin was significantly higher in AF patients compared to that in controls (*P* < 0.001). ROC analysis showed that higher serum adiponectin (>6.098 *μ*g/mL) had predictive efficacy for AF, with an area under the curve of 0.660 (95% confidence interval [CI]: 577–0.742). The results of multivariate logistic regression analysis showed that higher adiponectin was an independent predictor of AF in the overall participants (odds ratio [OR] 1.224, 95% CI 1.018–1.471, *P*=0.032). Subgroup analysis showed that higher adiponectin was independently associated with AF in women (OR 1.893, 95% CI 1.160–3.089, *P*=0.011) and in patients aged < 65 years (OR 1.453, 95% CI 1.023–2.064, *P*=0.037), but not in men or those aged ≥ 65 years.

**Conclusions:**

Higher serum adiponectin level was independently associated with higher odds for AF in women and in participants <65 years old, but not in men or those aged ≥65 years.

## 1. Introduction

Atrial fibrillation (AF) is a common arrhythmia in the elderly population and has been associated with higher risks of morbidity, mortality, and disability [1, 2]. Accumulating evidence from epidemiological studies has confirmed that obesity is an important risk factor for AF [3], but the key pathophysiological mechanisms underlying this association remain unknown. Adiponectin, one of the most abundant adipokines in human plasma, has been implicated in the pathogenesis of many chronic diseases due to its insulin-sensitizing, anti-inflammatory, and antioxidant effects [4–6]. Recent studies have related adiponectin with the risk of cardiovascular disease (CVD), and adiponectin is considered a potential biomarker for the risk of many CVDs [7–9], including AF. Previous prospective studies suggested that higher adiponectin could predict AF recurrence after catheter ablation in paroxysmal AF patients <65 years old [10]. However, lower plasma adiponectin was associated with a higher risk of major cardiovascular events after anticoagulation in women with AF, but not in men [11]. In view of the inconsistent results in previous studies, it could be hypothesized that the association between adiponectin and AF may be age- and gender-dependent. Additionally, atrial remodeling, autonomic imbalance, and inflammation have been recognized as important pathogenetic factors in AF [12–14]. However, the relationships between adiponectin and these pathogenetic factors in AF were rarely reported in the previous studies. Therefore, in this study, we aimed to evaluate the age- and gender-specific associations between adiponectin and AF in a case-control study. Moreover, the correlations between serum adiponectin and markers of atrial remodeling, autonomic imbalance, and inflammation were explored.

## 2. Materials and Methods

### 2.1. Study Population

We enrolled 84 consecutive patients with AF who underwent radiofrequency ablation at the Renmin Hospital of Wuhan University from March 2019 to October 2019. Age- and sex-matched individuals with normal sinus rhythm (NSR) were included as controls. Since plasma adiponectin levels can be affected by various comorbidities, patients with coronary artery disease, structural heart diseases, stroke, cardiac dysfunction (left ventricular ejection fraction [LVEF] < 50%), other arrhythmias (such as bradycardia, sick sinus syndrome, or ventricular arrhythmias), cancer, autoimmune diseases, hematological diseases, hepatorenal dysfunction (alanine aminotransferase [ALT] > 60 U/L, serum creatinine [SCr] > 120 umol/L), thyroid insufficiency, systemic acute diseases, and chronic infectious diseases were excluded. Data regarding demographic factors, clinical characteristics, CVD risk factors, blood biochemical parameters, echocardiography, and 24-hour dynamic electrocardiogram (ECG) results were collected.

### 2.2. Blood Sampling and Laboratory Analyses

Venous blood samples were collected from all participants, who were in a fasting state, the morning after admission. After centrifugation, the serum samples were stored at −80 centigrade until used for measurements. Fasting blood glucose, serum lipid, uric acid, and creatinine were determined with standard laboratory techniques using an automatic biochemical analyzer at the Central Laboratory of the Renmin Hospital of Wuhan University (Siemens Healthcare Diagnostics, Munich, Germany). Plasma adiponectin levels were determined with ELISA (Millipore, Billerica, Massachusetts, USA). High-sensitivity C-reactive protein (hs-CRP) was measured by nephelometry with a threshold of 0.01 mg/L. N-terminal pro-B-type natriuretic peptide (NT-proBNP) was measured with an electrochemical luminescence immunoassay analyzer (Roche, Basel, Switzerland) according to the manufacturer's instructions.

### 2.3. Echocardiography

All participants received transthoracic echocardiography with Color Doppler Echocardiography performed by the same skilled ultrasound physician. The left ventricular end-diastolic diameter (LVEDD) and left atrial anteroposterior diameter (LAD) were measured using standard M-mode echocardiography. LVEF was calculated with the modified Simpson method.

### 2.4. Holter Monitoring and Heart Rate Variability (HRV) Analysis

All participants completed 24-hour long-range 12-lead ECG recordings within 3 days of admission, and the corrected data were analyzed manually. The 24-hour mean heart rate and HRV were recorded. HRV analysis, which reflects the cardiac autonomic function, was performed using commercial software (H-Scribe Analysis system of American Mortara Company). The HRV indexes included both the time-domain parameters (the standard deviation of all normal sinus RR intervals [SDNN] and the root mean square successive difference [RMSSD]) and the frequency-domain parameters (high-frequency power [HF, 0.15–0.4 Hz], low-frequency power [LF, 0.04–0.15 Hz], and high-frequency/low-frequency [HF/LF] ratio). RMSSD and HF generally reflect cardiac parasympathetic nerve activity, while LF is related to cardiac sympathetic nerve activity. Accordingly, the LF/HF ratio reflects the balance between sympathetic and parasympathetic activities, with higher LF/HF values indicating increased sympathetic nerve excitability [15]. During analysis, only normal pulsations were measured, and patients with sinoatrial node dysfunction and a large number of abnormal rhythms (abnormal rhythm ≥ 5% effective rhythm) were excluded. In addition, the data segments for AF and out-of-period pulsation were excluded.

### 2.5. Statistical Analysis

The variance homogeneity test (Leven test) and normality test (F test) were performed to evaluate the distribution of each dataset. Continuous variables that conformed to the normal distribution were presented as the means and standard deviations; otherwise, medians (interquartile ranges) were applied. The two-sample *t*-test was used to compare the normal distribution variables between two groups, and the Mann–Whitney *U* test was used to compare the nonnormal distribution variables between two groups. For categorized variables, frequencies and rates were used, and a chi-square test was used for comparisons between two groups. Spearman correlation analysis was performed to evaluate the correlations between adiponectin and other factors. Binary logistic regression analysis was used to determine the factors associated with AF. Univariate logistic regression analysis was first used to evaluate the associations between clinical characteristics and AF. Variables with a correlation trend (*P* < 0.05) were included in the multivariate logistic regression model. A *P* < 0.05 was considered statistically significant. SPSS 22.0 software was used for statistical analysis.

## 3. Results

### 3.1. Characteristics of AF Patients and NSR Controls

Overall, 84 patients with AF and 84 people with NSR were included. The characteristics of the included participants are shown in Table 1. The levels of NT-proBNP and cardiac troponin I (cTnI) in the AF group were significantly higher than those in the NSR group (both *P* < 0.05). Among the echocardiographic parameters, there were significant differences in LAD (*P* < 0.001) and LVEF (*P* < 0.001) between the two groups. Differences in other characteristics were not significant between AF patients and NSR controls.

### 3.2. Association between Adiponectin and AF

**Figure 1:**
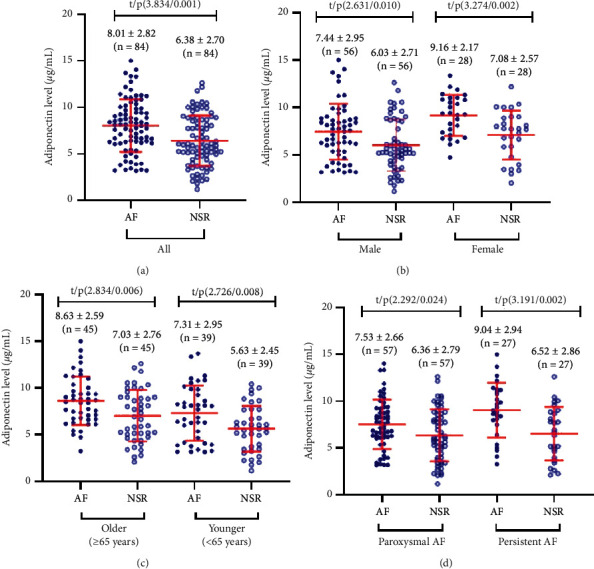
Serum adiponectin levels in patients with AF and controls with NSR: (a) scattergram comparing the serum adiponectin levels in the overall participants; (b) scattergram comparing the serum adiponectin levels according to the gender of the participants; (c) scattergram comparing the serum adiponectin levels according to the age of the participants; (d) scattergram comparing the serum adiponectin levels according to the type of AF.

The serum levels of adiponectin were significantly higher in AF patients than in NSR controls (*P* < 0.001, Figure 1(a)). Consistent results were found in subgroup analyses according to the age and sex of the participants, as well as the type of AF (Figures 1(b)–1(d)).

### 3.3. Association between Serum Adiponectin and AF Risk Factors

**Figure 2:**
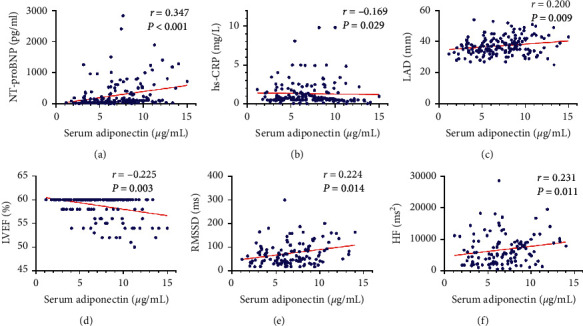
Correlation analyses between serum adiponectin and clinical parameters in the included participants: (a) correlation of adiponectin with NT-proBNP; (b) correlation of adiponectin with hs-CRP; (c) correlation of adiponectin with left atrium diameter (LAD); (d) correlation of adiponectin with LVEF; (e) correlation of adiponectin with root mean square successive difference (RMSSD); (f) correlation of adiponectin with high-frequency power (HF).

The results of Spearman correlation analysis showed that serum adiponectin was significantly correlated with NT-proBNP (*P* < 0.001, Figure 2(a)), hs-CRP (*P*=0.029, Figure 2(b)), LAD (*P*=0.009, Figure 2(c)), and LVEF (*P*=0.003, Figure 2(d)). Further, adiponectin showed positive correlations with RMSSD (*P*=0.014, Figure 2(e)) and HF (*P*=0.011, Figure 2(f)). Adiponectin levels were also significantly associated with gender, age, and body mass index (BMI), but not with LF and the LF/HF ratio (Table 2).

### 3.4. Association between Serum Adiponectin and AF Stratified by Age, Gender, and Type of AF

**Figure 3:**
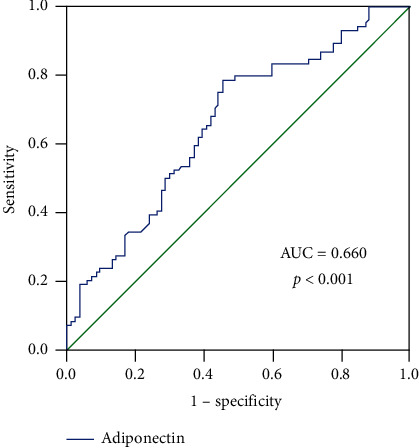
ROC analysis for the predictive efficacy of serum adiponectin for AF. As shown in the ROC analysis, with an optimal cut-off value of 6.098 *μ*g/mL, the sensitivity and specificity of serum adiponectin for predicting AF were 78.6% and 54.8%, with an AUC of 0.660 (95% CI: 577–0.742).

Univariate logistic regression analysis showed that NT-proBNP, LAD, LVEF, and adiponectin were related to the odds of AF (*P* < 0.05). Subsequent multivariate analysis showed that higher adiponectin was independently associated with AF in the overall participants (odds ratio [OR] 1.224, 95% confidence interval [CI]: 1.018–1.471, *P*=0.032; Table 3). Further subgroup analysis confirmed the independent association in women (OR 1.893, 95% CI: 1.160–3.089, *P*=0.011; Table 4), patients aged <65 years (OR 1.453, 95% CI 1.023–2.064, *P*=0.037; Table 5), and patients with paroxysmal AF (OR 1.229, 95% CI 1.005–1.503, *P*=0.045; Table 6), but not in men, those aged ≥65 years, or patients with persistent AF. The results of receiver operating characteristic (ROC) analysis showed a promising predictive efficacy of higher adiponectin for AF (Figure 3). With an optimal cut-off value of 6.098 *μ*g/mL, the sensitivity and specificity of serum adiponectin for predicting AF were 78.6% and 54.8%, with an area under the curve (AUC) of 0.660 (95% CI: 577–0.742).

## 4. Discussion

In this case-control study with AF patients and NSR controls, we found that the serum adiponectin levels in AF patients were significantly higher than those in NSR patients. This finding was consistent in subgroup analysis according to the age, gender, and AF type of the participants. Moreover, serum adiponectin was found to be correlated with markers of cardiac remodeling, inflammation, and cardiac autonomic function. Subsequent multivariate analysis showed a significant independent association between higher adiponectin levels and AF in the overall participants. Subgroup analysis showed a similar association in women, participants <65 years old, and patients with paroxysmal AF, but not in men, those aged ≥65 years, or patients with persistent AF. Taken together, our results showed that adiponectin is correlated with cardiac remodeling, inflammation, and cardiac autonomic function in AF patients. Moreover, the potential independent association between adiponectin and AF may be age- and sex-specific.

Previous studies showed that the association between serum adiponectin and the progression of CVD may have a “U” shape [16]. Similarly, the association between adiponectin and AF risk remains inconsistent [17]. Two cross-sectional studies showed that, compared to the levels in people with sinus rhythm, serum adiponectin levels were significantly higher in persistent AF patients [18], but significantly lower in patients with paroxysmal AF [19]. These conflicting results may be explained by the potential influences of sex, age, BMI, or comorbidities in patients with these associations. One of the strengths of our study is that we included age- and sex-matched AF patients and NSR controls, and we strictly excluded patients with comorbidities that may affect the serum adiponectin. Our results showed that higher adiponectin was independently associated with AF, and the association remained in women, participants <65 years old, and patients with paroxysmal AF. Currently, it remains unknown whether higher adiponectin is only a marker or an active participant in the pathogenesis of AF. Previous studies showed that the binding of adiponectin to its receptor, an increase in adiponectin resistance, and the compensatory secretion of adiponectin may be involved in AF. Further research is needed to evaluate the potential mechanisms underlying the association between higher adiponectin and AF.

Atrial remodeling, inflammation, and autonomic dysfunction have been recognized as key pathogenetic factors for AF. It has been suggested in previous studies that adiponectin may be associated with these mechanisms. Ybarra et al. [20] found that adiponectin was negatively correlated with left atrial size, which could inhibit atrial interstitial fibrosis and reverse atrial remodeling [18]. However, our study showed a positive correlation between adiponectin and LAD. We speculate that adiponectin may present with a compensatory increase in patients with AF as a protective hormone. Adiponectin is also considered a powerful “anti-inflammatory factor,” which acts by binding to adiponectin receptors on the membrane of target cells [21]. Studies have shown that adiponectin can inhibit the expression of scavenger receptors, vascular adhesion molecules, and proinflammatory cytokines (TNF-*α*, IL-6, IL-1*β*) in various tissues [22] and inhibit cardiac inflammation and progression by upregulating anti-inflammatory cytokines [23]. Our study also showed a significant negative correlation between adiponectin and hs-CRP, suggesting that adiponectin may have a protective effect in AF patients via its anti-inflammatory effect. In addition, previous studies have also suggested a relationship between adiponectin and autonomic function. In patients with type 2 diabetes, higher adiponectin was reported to be associated with more favorable cardiac autonomic function [24]. Moreover, adiponectin was reported as a positive horizontal and longitudinal predictor of parasympathetic nerve activity in women [25]. Our study showed that adiponectin was positively correlated with RMSSD and HF, which suggests that higher serum adiponectin may be associated with an increased cardiac parasympathetic activity. The implications of this association on the pathogenesis of AF deserve further evaluation.

Previous studies have suggested age and gender differences in the relationship between adiponectin and AF [10, 11]. In our study, although the ROC curve analysis showed that an adiponectin level ≥6.098 ng/mL had a predictive value for the occurrence of AF in the overall participants, the association between adiponectin and AF was mainly observed in women and those aged <65 years. Previous studies showed that circulating BNP can affect plasma adiponectin levels [26, 27] and may explain the controversial results for the relationship between circulating adiponectin levels and the prediction of CVD [28]. It could be inferred that there is a compensatory increase of adiponectin in elderly patients with AF to reduce cardiac remodeling and inflammation. Several possible mechanisms may explain the gender and age differences in the role of adiponectin in AF. Firstly, it has been suggested that the androgen testosterone can inhibit adiponectin secretion by adipocytes [29]. Adiponectin may have different metabolic effects in men. Secondly, with the increase of age, adiponectin receptors may be downregulated or resistance may develop [30], which can cause positive feedback. Although adiponectin levels are higher in elderly individuals, the elevated levels may not have corresponding beneficial effects because the incidence of AF gradually increases with age. The potential age- and sex-specific association between adiponectin and AF should be validated in large-scale studies.

Some limitations of the current study should be noted. Firstly, the sample size of the current study was limited, and the findings should be further verified in large-scale studies. Moreover, the current analysis was cross-sectional. Large-scale prospective cohort studies are needed to determine the independent association between adiponectin and AF risk according to the age and gender of the participants. In addition, it remains unclear whether elevated adiponectin is a cause or a consequence of AF. Furthermore, in this study, only hs-CRP was measured as an indicator of systematic inflammation and other inflammatory factors such as IL-6, IL-1 *β*, and TNF-*α* were not investigated. The associations between adiponectin and these inflammatory factors in AF remain to be evaluated. Finally, other important factors that affect plasma adiponectin levels, such as body fat content and waist circumference, were not measured in our study. Variations in these factors may confound the association between adiponectin and AF in our study population.

## 5. Conclusion

The present study suggested that adiponectin is correlated with cardiac remodeling, inflammation, and cardiac autonomic function in AF patients. Moreover, the potential independent association between adiponectin and AF may be age- and sex-specific. These results should be confirmed in large-scale prospective studies, and the mechanisms underlying the association between adiponectin and AF should be further investigated in future studies.

## Figures and Tables

**Table 1:** Clinical characteristics of patients with AF and controls with NSR.

Clinical characteristics	AF (*n* = 84)	NSR (*n* = 84)	*P* value
Age (years)	61.3 ± 10.3	60.7 ± 10.8	0.705
Men (*n*/%)	56 (67)	56 (67)	1.000
Body mass index (kg/m^2^)	24.8 ± 3.3	24.2 ± 3.2	0.400
Hypertension (*n*/%)	40 (48)	44 (52)	0.537
Diabetes mellitus (*n*/%)	10 (12)	7 (8)	0.443
Smoking (*n*/%)	19 (23)	28 (33)	0.314
Drinking (*n*/%)	16 (19)	11 (13)	0.294
ACEI/ARB (*n*/%)	26 (31)	22 (26)	0.738
TZDs (*n*/%)	2 (2.4)	3 (3.6)	0.652
Paroxysmal AF (*n*/%)	57 (68)	—	—
Persistent AF (*n*/%)	27 (32)	—	—
NT-proBNP (pg/ml)	321.2 (113.1–716.7)	55.5 (25.6–110.2)	**<0.001**
cTnI (ng/ml)	0.006 (0.006–0.125)	0.006 (0.006–0.006)	**0.001**
Hs-CRP (mg/L)	0.68 (0.50–1.24)	0.81 (0.50–1.89)	0.643
Fasting glucose (mmol/L)	4.96 (4.57–5.78)	4.88 (4.44–5.87)	0.951
Uric acid (*μ*mol/L)	384.3 ± 92.1	400.5 ± 123.7	0.339
Total cholesterol (mmol/L)	4.19 ± 0.81	4.52 ± 0.89	**0.015**
Triglycerides (mmol/L)	1.53 (1.10–2.28)	1.88 (1.16–2.69)	0.090
HDL-cholesterol (mmol/L)	1.17 ± 0.45	1.13 ± 0.33	0.499
LDL-cholesterol (mmol/L)	2.36 ± 0.68	2.59 ± 0.82	**0.048**
eGFR (mL/min)	93.7 ± 19.4	95.8 ± 19.5	0.496
Left atrium diameter (mm)	40.2 ± 5.6	34.4 ± 4.1	**<0.001**
LVEDD (mm)	44.9 ± 5.5	44.0 ± 3.6	0.199
LVEF (%)	57.7 ± 3.1	59.9 ± 1.9	**<0.001**

Continuous variables are presented as mean ± SD or median and IQR. Categorized variables are presented as number (percentage). *P* values < 0.05 are indicated in bold. AF, atrial fibrillation; NSR, normal sinus rhythm; ACEI, angiotensin-converting enzyme inhibitor; ARB, angiotensin type II receptor blockers; TZD, thiazolidinedione; NT-proBNP, N-terminal probrain natriuretic peptide; cTnI, cardiac troponin I; hs-CRP, high-sensitivity C-reactive protein; HDL, high-density lipoprotein; LDL, low-density lipoprotein; eGFR, estimated glomerular filtration rate; LVEDD, left ventricular end-diastolic dimension; LVEF, left ventricle ejection fraction.

**Table 2:** Correlation between plasma adiponectin concentration and other parameters.

Variable	*r*	*P* value
Female	0.256	**0.001**
Age	0.279	**<0.001**
Body mass index	−0.234	**0.015**
NT-proBNP	0.347	**<0.001**
Hs-CRP	−0.169	**0.029**
Left atrium diameter	0.200	**0.009**
LVEF	−0.225	**0.003**
rMSSD	0.224	**0.014**
LF	0.071	0.439
HF	0.231	**0.011**
LF/HF ratio	−0.100	0.275

*P* values < 0.05 are indicated in bold. NT-proBNP, N-terminal probrain natriuretic peptide; hs-CRP, high-sensitivity C-reactive protein; LVEF, left ventricle ejection fraction; rMSSD, root mean square successive difference; LF, low-frequency component; HF, high-frequency component.

**Table 3:** Univariate and multivariate logistic regression analysis for the independent predictors of AF.

Variable	Univariate analysis		Multivariate analysis	
	OR (95% CI)	*P* value	OR (95% CI)	*P* value
NT-proBNP	1.011 (1.006–1.015)	**<0.001**	1.009 (1.004–1.014)	**<0.001**
Total cholesterol	0.634 (0.436–0.921)	**0.017**	0.418 (0.118–1.478)	0.176
Triglyceride	0.792 (0.624–1.005)	**0.049**	1.010 (0.658–1.549)	0.964
LDL-cholesterol	0.657 (0.431–1.001)	**0.041**	1.358 (0.398–4.636)	0.625
Left atrium diameter	1.294 (1.185–1.412)	**<0.001**	1.149 (1.030–1.281)	**0.013**
LVEF	0.575(0.442–0.748)	**<0.001**	0.856 (0.643–1.138)	0.284
Adiponectin	1.239 (1.101–1.393)	**<0.001**	1.224 (1.018–1.471)	**0.032**

*P* values <0.05 are indicated in bold. OR, odds ratio; CI, confidence interval; AF, atrial fibrillation; NT-proBNP, N-terminal probrain natriuretic peptide; LDL, low-density lipoprotein; LVEF, left ventricle ejection fraction.

**Table 4:** Univariate and multivariate logistic regression analysis for the independent predictors of AF stratified by gender.

Variable	Univariate analysis		Multivariate analysis	
	OR (95% CI)	*P* value	OR (95% CI)	*P* value
*Female*				
NT-proBNP	1.007 (1.002–1.012)	**0.008**	1.008 (1.000–1.015)	**0.043**
hs-CRP	0.532 (0.274–1.034)	**0.036**	0.764 (0.451–1.293)	0.316
eGFR	0.963 (0.930–0.998)	**0.039**	1.001 (0.944–1.061)	0.977
Left atrium diameter	1.238 (1.082–1.416)	**0.002**	1.169 (0.933–1.463)	0.174
LVEF	0.497 (0.273–0.903)	**0.022**	0.457 (0.116–1.876)	0.283
Adiponectin	1.458 (1.120–1.896)	**0.005**	1.893 (1.160–3.089)	**0.011**

*Male*				
NT-proBNP	1.014 (1.008–1.021)	**<0.001**	1.012 (1.005–1.019)	**0.001**
Uric acid	0.996 (0.993–1.000)	**0.046**	0.991 (0.984–0.998)	**0.014**
Left atrium diameter	1.345 (1.194–1.515)	**<0.001**	1.228 (1.058–1.425)	**0.007**
LVEF	0.599 (0.450–0.798)	**<0.001**	0.760 (0.528–1.096)	0.142
Adiponectin	1.195 (1.039–1.374)	**0.012**	0.869 (0.664–1.137)	0.306

*P* values <0.05 are indicated bold. OR, odds ratio; CI, confidence interval; AF, atrial fibrillation; NT-proBNP, N-terminal probrain natriuretic peptide; hs-CRP, high-sensitivity C-reactive protein; eGFR, estimated glomerular filtration rate; LVEF, left ventricle ejection fraction.

**Table 5:** Univariate and multivariate logistic regression analysis for the independent predictors of AF stratified by age.

Variable	Univariate analysis		Multivariate analysis	
	OR (95% CI)	*P* value	OR (95% CI)	*P* value
*Age < 65 years*				
NT-proBNP	1.021 (1.008–1.034)	**0.002**	1.018 (1.004–1.033)	**0.015**
Total cholesterol	0.517 (0.265–1.008)	**0.045**	0.346 (0.085–1.408)	0.138
Left atrium diameter	1.308 (1.141–1.498)	**<0.001**	1.277 (1.025–1.589)	**0.029**
LVEF	0.465 (0.257–0.841)	**0.011**	0.402 (0.136–1.189)	0.100
Adiponectin	1.259 (1.054–1.504)	**0.011**	1.453 (1.023–2.064)	**0.037**

*Age ≥ 65 years*				
NT-proBNP	1.009 (1.004–1.014)	**0.001**	1.008 (1.002–1.014)	**0.005**
Left atrium diameter	1.328 (1.171–1.507)	**<0.001**	1.231 (1.057–1.433)	**0.007**
LVEF	0.613 (0.460–0.817)	**0.001**	0.903 (0.654–1.248)	0.537
Adiponectin	1.253 (1.060–1.481)	**0.008**	1.195 (0.925–1.542)	0.173

*P* values <0.05 are indicated in bold. OR, odds ratio; CI, confidence interval; AF, atrial fibrillation; NT-proBNP, N-terminal probrain natriuretic peptide; LVEF, left ventricle ejection fraction.

**Table 6:** Univariate and multivariate logistic regression analysis for the independent predictors of AF stratified by type of AF.

Variable	Univariate analysis		Multivariate analysis	
	OR (95% CI)	*P* value	OR (95% CI)	*P* value
*Paroxysmal AF*				
NT-proBNP	1.010 (1.005–1.034)	<0.001	1.009 (1.003–1.015)	**0.002**
Total cholesterol	0.490 (0.301–0.797)	0.004	0.279 (0.087–1.021)	0.054
LDL-cholesterol	0.513 (0.302–0.872)	0.014	1.422 (0.419–4.828)	0.572
Left atrium diameter	1.284 (1.150–1.438)	<0.001	1.164 (1.019–1.328)	**0.025**
LVEF	0.666 (0.512–0.867)	0.002	0.940 (0.738–1.196)	0.614
Adiponectin	1.173 (1.019–1.351)	0.027	1.229 (1.005–1.503)	**0.045**

*Persistent AF*				
NT-proBNP	1.018 (1.006–1.030)	0.003	1.018 (1.001–1.035)	**0.038**
Left atrium diameter	1.371 (1.161–1.620)	<0.001	1.305 (0.983–1.733)	**0.066**
LVEF	0.649 (0.474–0.887)	0.007	1.311 (0.722–2.380)	0.374
Adiponectin	1.349 (1.092–1.667)	0.005	1.383 (0.822–2.328)	0.222

*P* values <0.05 are indicated in bold. OR, odds ratio; CI, confidence interval; AF, atrial fibrillation; NT-proBNP, N-terminal probrain natriuretic peptide; LDL, low-density lipoprotein; LVEF, left ventricle ejection fraction.

## Data Availability

The data that support the findings of this study are available from the Renmin Hospital of Wuhan University. But restrictions apply to the availability of these data, which were used under license for the current study and so are not publicly available. The data are however available from the authors upon reasonable request and with the permission of the Renmin Hospital of Wuhan University.
